# A New Mathematical Model of Functionally Graded Porous Euler–Bernoulli Nanoscaled Beams Taking into Account Some Types of Nonlinearities

**DOI:** 10.3390/ma15207186

**Published:** 2022-10-15

**Authors:** A. V. Krysko, I. V. Papkova, A. F. Rezchikov, V. A. Krysko

**Affiliations:** 1 Lavrentyev Institute of Hydrodynamics of SB RAS, Lavrentyev Av., 15, Novosibirsk 630090, Russia; 2 V.A. Trapeznikov Institute of Control Sciences of Russian Academy of Sciences, Profsouznaya 65, Moscow 117997, Russia

**Keywords:** modified couple stress theory, porous FGM beam, theory of plasticity, geometric nonlinearity, method of variable elasticity parameters, method of establishment

## Abstract

A new mathematical model of flexible physically (FN), geometrically (GN), and simultaneously physically and geometrically (PGN) nonlinear porous functionally graded (PFG) Euler–Bernoulli beams was developed using a modified couple stress theory. The ceramic phase of the functionally material was considered as an elastic material. The metal phase was considered as a physically non-linear material dependent on coordinates, time, and stress–strain state, which gave the opportunity to apply the deformation theory of plasticity. The governing equations of the beam as well as boundary and initial conditions were derived using Hamilton’s principle and the finite difference method (FDM) with a second-order approximation. The Cauchy problem was solved by several methods such as Runge–Kutta from 4-th to 8-th order accuracy and the Newmark method. Static problems, with the help of the establishment method, were solved. At each time step, nested iterative procedures of Birger method of variable elasticity parameters and Newton’s method were built. The Mises criterion was adopted as a criterion for plasticity. Three types of porosity-dependent material properties are incorporated into the mathematical modeling. For metal beams, taking into account the geometric and physical nonlinearity, the phenomenon of changing the boundary conditions, i.e., constructive nonlinearity (CN) was found.

## 1. Introduction

Mathematical modeling of functionally graded porous size-dependent structural members of devices is nowadays a topic of significant interest in engineering. Ceramic metal functionally graded materials (FGMs) have been extensively used in aerospace engineering where high strength and excellent heat insulation materials are desired. In paper [1], the thermodynamic behavior of the thermal protection system (TPS) using bolted joints made up of porous ZrO_2_/(ZrO_2_ þ Ni) FGMs was investigated by finite-element (FE) modeling. The effects of the preload on the thermomechanical behavior and service reliability of the bolted joint were numerically analyzed in detail by ABAQUS codes. Functionally graded materials (FGMs) are one of the most interesting objects that have been recently utilized to conceive modern Micro/Nanoelectromechanical systems (MEMS/NEMS) [2,3]. Porous functionally graded (FG) micro/nanobeams, micro/nanobeams are the basis structural members for designing the different complexity of the MEMS and NEMS. Currently, a large number of contributions have been published on the study of the size-dependent behavior of beams, plates at micro- and nano-scale to create new systems, such as piezoelectric devices for biomedicine applications [4], resonators [5], and resonant accelerometers [6]. MEMS/NEMS resonators based on suspended beams are one of the most common micro/nanodevices [7]. The latter composite micro/nanostructures are often subjected to static and dynamic loads. The stress–strain state of structural members and their mechanical characteristics significantly impact on the work of the MEMS/NEMS resonators. 

Recently, Fan et al. [8] studied the geometrically nonlinear oscillations of microplates with and without a central notch, made of a porous functionally graded material (PFGM), taking into account the size dependence of the pair stress. To this end, a new power function was used that could simultaneously take into account the influence of the material graded and the porosity. Three different porosity distribution models were considered, namely uniform porosity (U-PFGM), reduced porosity from the top and bottom surfaces to the center (X-PFGM), and increased porosity from the top and bottom surfaces to the center (O-PFGM). Young’s modulus, Poisson’s ratio, and material density varied depending on the thickness position and length of the plate corresponding to each porosity distribution pattern. Some studies consider various approximations of the porous structure.

Hou et al. [9] analyzed the bending and buckling analysis of microbeam with the uniform and non-uniform cylindrical cross-section based on the classical beam theory and the modified couple stress theory. The nonlinear effect of the Von Kármán theory was taken into account to study the large defection effect on the buckling of microtubes. Porous functionally material nanotubes were taken into account by two-dimensional trigonometric dependencies. Li et al. [10] presented a novel model of the nonlinearized bending behaviors of two-dimensionally FG beams based on the Euler–Bernoulli beam kinematic theory. For modeling two-dimensionally FG materials, Young’s modulus varying along the length or axial direction was taken into account by an exponential distribution function, and Young’s modulus varying along the thickness direction was taken into account by a power law function. Tang et al. [11] analyzed free vibration analysis of FG Euler–Bernoulli nanobeam based on non-local elasticity. For FG, the nanobeam is assumed to change the porosity of the structure in two coordinates using a power function. In the works cited above, the connection between the inhomogeneity of the material and the stress–strain state beam was not taken into account. Dehrouyeh-Semnani [12] investigated the non-linear response of a functionally shear deformable slightly curved beam to the thermal loading. The first order shear deformation theory of beam in conjunction with neutral surface concept was implemented. An exact analytical solution was suggested for the beam with clamped ends. Some verification studies were carried out for the system without initial curvature. The literature review below shows that a great number of beam theories are applied to the analysis behaviors of porous functionally graded (PFG) nanobeams. Beam theories are usually divided into three main groups as follows: classical beam theory (CBT) or Euler–Bernoulli beam theory, first-order shear deformation (FSDT) beam theory or Timoshenko beam theory, and the third-order shear deformation theory (TSDT) or Sheremetev–Pelekh–Reddy–Levinson beam theory. Krysko et al. [13] presented a review of mathematical models based on the kinematic hypotheses of Euler–Bernoulli, Timoshenko, and Sheremetev–Pelekh–Reddy. Some remarks should be made here: the Reddy hypothesis was proposed 27 years later than the same hypothesis of the Ukrainian scientists Sheremetiev and Pelekh [14]. Usually at designing micro- and nanostructures different beam models are used in combination with theories that take into account the various size-dependent effects, for example, Eringen’s nonlocal theory [15], strain gradient theory [16], modified couple stress theory [17], etc. Eltaher et al. [18] studied the mechanical bending and vibration of functionally graded nanobeams using finite elements according to Euler–Bernoulli beam theory. The effect of nanoscale was taken into account by the nonlocal continuum theory. The material gradation of constituents was described by a power function through the thickness of nanobeam. Mirjavadiet et al. [19] investigated the buckling and nonlinear vibration of PFG nanobeam based on the Euler–Bernoulli beam theory. The nonlocal Eringen’s theory was used to study the size effects. Shafiei et al. [20] analyzed the vibration behavior of the two-dimensional functionally graded (2D-FG) nano and microbeams which are made of two kinds of porous materials for the first time, taking into account Timoshenko beam theory, the Eringen’s nonlocal elasticity and the modified couple stress theories in case of nano and microbeams. Gui-Lin She al. [21] investigated the wave propagation behaviors of FG materials porous nanobeams based on Reddy’s higher order shear beam deformation theory in conjunction with the non-local strain gradient theory. Upon solving an eigenvalue problem, the analytic dispersion relation is received. The Euler–Bernoulli and Timoshenko beam models are also presented. The influences of non-local parameter, strain gradient parameter, power law index and porosity volume fraction on the wave propagation are discussed in detail. Krysko et al. [22] tested a size-dependent model of a Sheremetev–Pelekh–Reddy–Levinson micro-beam and validated using the couple stress theory, taking into account large deformations. A novel scenario of transition from regular to chaotic vibrations of the size-dependent Sheremetev–Pelekh model, following the Pomeau–Manneville route to chaos, is also detected and illustrated, among others. She et al. [23] studied the statics of FG beams for the prediction of thermal buckling of beams and their postbuckling behaviors based on taking into account Euler–Bernoulli, Timoshenko and various higher-order shear beam deformation theories. The following higher-order shear deformation beam theories have been applied: TSDBT (from Touratier [24]); HSDBT (from Soldatos [25]); ESDBT (from Karama et al. [26]); ASDBT (from Aydogdu [27]); HBTM1 (from Mantari et al. [28]); HBTM2 from Mantari et al. [29]); HBTAT1 (from Akavci et al. [30]); HBTV1 (from Viola et al. [31]); HBTV2 (from Meiche et al. [32]). Belarbi et al. [33] investigated the size-dependent bending and buckling analysis of FG nanobeams using finite element approach and based on nonlocal elasticity theory using a novel parabolic shear deformation beam theory. Mechanical properties of the FG nanobeams are considered to vary over the thickness based on the power law. The robustness and the reliability of the developed finite element model were validated by comparing the author’s results with various analytical solutions available in the literature. Moreover, a parametric study was conducted to bring out the influence of various parameters such as length-to-height ratio, nonlocal parameter, boundary conditions and power law index. Akbas et al. [34] presented a dynamic analysis of a simply supported PFG microbeam subjected to a moving load. The porosity distributions of the microbeam in the thickness direction were taken into account according to power law distribution with different porosity models. The governing equations are obtained by the Lagrange procedure taking into account Bernoulli–Euler beam model and modified couple stress theories. The effects of porosity coefficient, porosity distribution, material distribution, and length scale parameter on the dynamic responses of functionally porous microbeams are being investigated. Van Hieu Dang & Quang-Chan Do [35] developed a microbeam model based on the Euler–Bernoulli beam theory and the nonlocal strain gradient theory to study nonlinear vibration and stability of a functionally graded porous microbeam under electrostatic actuation. The porosity distributions of the microbeam change along thickness direction according to simple power law distribution. Effects of the material length scale parameter, the nonlocal parameter, the porosity distribution factor on nonlinear vibration and stability of the functionally graded porous microbeam are discussed. Quoc-Hoa Pham et al. [36] presented higher-order shear beam deformation theory taking into account the strain gradient theory to study the dynamic instability of magnetically embedded FGP nanobeams. Nanobeams with elastic foundation (EF) subjected to an axially oscillating load are analyzed. Hosseini et al. [37] presented a higher-order shear beam deformation theory taking into account the strain gradient theory to study the dynamic instability of magnetically embedded FGP nanobeams. Nanobeams with elastic foundation (EF) subjected to an axially oscillating load were analyzed. Bolotin’s method is employed to determine the instability region of FGP nanobeams. The influences of magnetic potential, small-scale parameter, porosity coefficient, stiffness foundation, boundary conditions (BCs) on the dynamic instability of nanobeams were studied in detail. Recently, the scientific community has been taking the first steps to take into account physical nonlinearity for functionally graded porous structures. Nguyen et al. [38] studied nonlinear bending of elasto-plastic FG ceramic-metal beams subjected to nonuniform distributed loads by the finite element method. Based on Euler–Bernoulli beam theory, a non-linear finite element formulation, taking the effect of plastic deformation into account, was derived. The influence of the material distribution and plastic deformation on the nonlinear behavior of the beam are investigated for various boundary conditions. Nikbakht et al. [39] reviewed the majority of publications on the optimization of FG structures. In addition to FG beams, plates and shells, various structures such as tubes, implants, rotating disks, sports instruments, etc. were researched. It should be noted that in the above review [39], the authors also refer to this single work [38].

Summing up the above review, we note the lack of research on porous functionally graded nanoscaled beam structures, taking into account the elastic-plastic properties of the material and geometric nonlinearity.

In this paper, a mathematical model of statics and nonlinear dynamics of flexible physically nonlinear porous functionally graded (metal/ceramic) nanoscaled Euler–Bernoulli beams was built for the first time. Methods for their study were developed. Some different porosity distributions were considered. The influences of the length scale parameter l, the modified couples stress theory on the stress–strain state of Euler–Bernoulli beams are studied in detail.

The paper is organized in the following way. In Section 2, a new mathematical model of porous functionally graded (PFG) nanoscaled Euler–Bernoulli beams taking physical nonlinearity (FN), geometrically nonlinearity (GN) and simultaneously physical and geometric nonlinearity (FGN) were developed. Solution methods were discussed in Section 3. Numerical results regarding nanobeams were reported in Section 4. Results were reported and discussed. Section 5 includes the concluding remarks.

## 2. Problem Formulation

**Figure 1:**
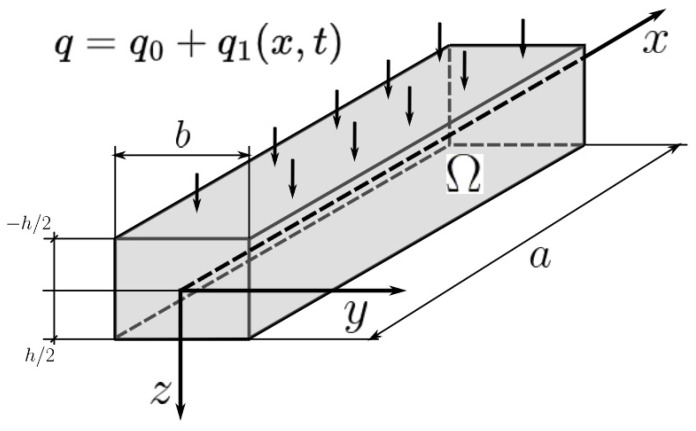
The investigated beam.

To construct a new mathematical model of statics and dynamics of porous functionally graded (PFG) Euler–Bernoulli nanoscaled beams, taking into account the (FN) the beam nanostructure length—*a*, thickness h and width *b* is presented in the form of a three-dimensional (3D) region Ω: $\Omega =\left\{x\in \left[0;a\right];\;y\in \left[-b/2;\;b/2\right];\;z\in \left[-h/2;\;h/2\right]\right\}$, 0≤t≤∞ of space *R*^3^ in the Cartesian rectangular coordinate system (Figure 1). The origin of coordinates is located in the center of the beam in its middle surface, the 𝑥, 𝑦 axes are parallel to the sides of the beam, and the 𝑧 axis is directed down. Further we will consider *b* = 1.

We assume that the beam material is porous and FG. The nanobeam is subjected to the action of a transverse sign-variable load, $q_{0}$—where stands for the load amplitude. The hypotheses and assumptions were applied for constructing a mathematical model:

1. Accepted classical beam theory (CBT) or Euler–Bernoulli beam theory:

(1) $$\varepsilon_{xx}^{b}=-z\frac{\partial^{2}w}{\partial x^{2}},$$

2. Deformations along the axis—*x* are governed by the formula: $\varepsilon_{xx}=\varepsilon_{xx}^{b}+\varepsilon_{xx}^{m}$. Deformations in the beam middle surface are taken into account in the form of von Kármán: $\varepsilon_{xx}^{m}=\frac{\partial u}{\partial x}+\frac{1}{2}{\left(\frac{\partial w}{\partial x}\right)}^{2}$, where *u* = *u(x)*, *w = w(x)*—displacement and deflection along the axis *x* respectively. This is a geometric nonlinearity (GN);

3. Let the material properties of nanobeam such as the Young’s modulus, Poisson’s ratio and density be defined by using the following ratios:

(2) $$E\left(x,z,e_{0},e_{i},t\right)=\left(E_{c}-E_{m}\left(x,z,e_{0},e_{i},t\right)\right)\cdot \varphi \left(z\right)+E_{m}\left(x,z,e_{0},e_{i},t\right)-\;-\left(E_{c}-E_{m}\left(x,z,e_{0},e_{i},t\right)\right)\cdot \psi \left(z\right)\cdot \frac{\Gamma }{2},$$

(3) $$\nu \left(x,z,e_{0},e_{i},t\right)=\left(\nu_{c}-\nu_{m}\left(x,z,e_{0},e_{i},t\right)\right)\cdot \varphi \left(z\right)+\nu_{m}\left(x,z,e_{0},e_{i},t\right)-\;-\left(\nu_{c}-\nu_{m}\left(x,z,e_{0},e_{i},t\right)\right)\cdot \psi \left(z\right)\cdot \frac{\Gamma }{2},$$

(4) $$\rho \left(x,z,e_{0},e_{i},t\right)=\left(\rho_{c}-\rho_{m}\left(x,z,e_{0},e_{i},t\right)\right)\cdot \varphi \left(z\right)+\rho_{m}\left(x,z,e_{0},e_{i},t\right)-\;-\left(\rho_{c}-\rho_{m}\left(x,z,e_{0},e_{i},t\right)\right)\cdot \psi \left(z\right)\cdot \frac{\Gamma }{2},$$

where Γ—represents the porosity index, *E_c_*, *E_m_*, *ν_c_*, *ν_m_*, *ρ_c_*, *ρ_m_*—the Young’s modulus, Poisson’s ratio, and density associated with the ceramic and metal phases FGM. If Γ = 0, then no pores. It should be noted, that in works [7,8] *E_m_*, *ν_m_*, *ρ_m_* are constants. For ceramics *E_c_*, *ν_c_*, *ρ_c_* are indeed constant because there is practically no yield strength. For metal *E_m_*, *ν_m_*, *ρ_m_* are not constants, but functions that depend on coordinates *x*, *z*, and bulk deformation—$e_{0},$ deformation intensity—$e_{i}$ and time *t*. Therefore, in this work, we assume that $E_{m}\left(x,\;z,e_{0},\;e_{i},t\right),\;\nu_{m}\left(x,\;z,\;e_{0},e_{i},t\right)$ are the functions of the variables $\left(x,\;z,\;e_{0},e_{i},t\right)$ and the Young’s modulus $E\left(x,\;z,e_{0},\;e_{i},t\right)$, the Poisson’s ratio $\nu \left(x,\;z,e_{0},\;e_{i},t\right)$ and the density of the beam material $\rho \left(x,\;z,e_{0},\;e_{i},t\right)$ are the functions of the variables $\left(x,\;z,e_{0},\;e_{i},t\right)$ also. This approach is new in the theory of functionally graded structures. Such a representation according to the Birger’s method of variable elasticity parameters [40] gives the ability to take into account the physical nonlinearity of the material FG beams. Problem statement in the form (2–4) is used for the first time. For the proposed mathematical model, the presence of material inhomogeneity is important. There are various ways to account and changes of material inhomogeneity. So, for example, if the Young’s modulus *E* is a function of x,z, then this corresponds to a inhomogeneous physically linear material, and if the Young’s modulus *E* is a function of $e_{0},e_{i}$ ($e_{0}\left(x,z\right)$—bulk deformation, $e_{i}\left(x,z\right)$—deformation intensity), then this corresponds to a inhomogeneous before deformation, physically nonlinear material. Note that in the proposed mathematical model, Young’s modulus is taken into account as a function *E*(*x, z*, $e_{0},$ $e_{i}$) defined and non-linear connected in a way with the deformed state at the considered point.

On these assumptions the method of variable elasticity parameters for solving nonlinear elastic and elastic-plastic problems is based. We apply Hencky’s theory of small elastic–plastic deformations. The ratio of the theory of small elastic–plastic deformations is based on the assumption that at each point of the body, the loading path is straight. This limitation is quite strict. It is known that if unloading does not occur at any point of the body, then this theory coincides with the theory of an elastic physically nonlinear body.

Later Budiansky [41], under the assumption of the singularity of surface loading, proved that Hencky’s deformation theory does not contradict the Drucker postulates of plasticity [42] (it is physically consistent) when the trajectory of motion deviates from straight lines. Ohashi, Y et al. [43] showed that in the case of an ideal elastoplastic diagram $\sigma_{i}\left(e_{i}\right)$ without hardening, when using the von Mises plasticity criterion, Budyansky’s criterion for the applicability of the deformation theory is satisfied. Thanks to these works, the theoretically based application of the deformation theory was significantly expanded;

4. Physical nonlinearity (FN) is introduced using the deformation theory of plasticity. The Young’s modulus $E\left(x,\;z,\;e_{0},e_{i},t\right)$ and the Poisson’s ratio $\nu \left(x,\;z,\;e_{0},e_{i},t\right)$ are coupled with the shear modulus $G\left(x,\;z,\;e_{0},e_{i},t\right)$ and volumetric modulus $K\left(x,\;z,\;e_{0},e_{i},t\right)$ via the following relations:

(5) $$E\left(x,\;z,e_{0},\;e_{i},t\right)=\frac{9K\left(x,\;z,\;e_{0},e_{i},t\right)G\left(x,\;z,e_{0},\;e_{i},t\right)}{3K\left(x,\;z,e_{0},\;e_{i},t\right)+G\left(x,\;z,\;e_{0},e_{i},t\right)},\;\nu \left(x,\;z,e_{0}\;e_{i},t\right)=\frac{3K\left(x,\;z,e_{0},\;e_{i},t\right)-2G\left(x,\;z,\;e_{i},t\right)}{3K\left(x,\;z,e_{0},\;e_{i},t\right)+G\left(x,\;z,e_{0},\;e_{i},t\right)}.$$

By the deformation theory of plasticity, the shear modulus is determined through the following formula

(6) $$G\left(x,\;z,e_{0},\;e_{i},t\right)=\frac{1}{3}\frac{\sigma_{i}\left(e_{i}\right)}{e_{i}},$$

where $\sigma_{i}\left(e_{i}\right)$ is the stress intensity dependence from deformation intensity, which can be determined experimentally, given numerically or analytically. The following expression determines intensity deformations:

(7) $$e_{i}=\frac{\sqrt{2}}{3}{\left[e_{xx}^{2}+e_{zz}^{2}+{\left(e_{xx}-e_{zz}\right)}^{2}\right]}^{\frac{1}{2}}.$$

According to the Euler–Bernoulli, hypotheses $e_{zz}$ should be equal to zero. However, at the study of elastic-plastic deformations according to the deformation theory of plasticity $e_{zz}$ is an equivalent component and it can be determined from the condition of a plane stress state $\left(\sigma_{zz}=0\right)$:

(8) $$e_{zz}=-\frac{\nu }{1-\nu }e_{xx}$$

Then $e_{i}$ will take the form:

(9) $$e_{i}=\frac{2}{3}{\left(1+\frac{\nu }{1-\nu }+{\left(\frac{\nu }{1-\nu }\right)}^{2}\right)}^{1/2}[\frac{\partial u}{\partial x}+\frac{1}{2}{\left(\frac{\partial w}{\partial x}\right)}^{2}-z\frac{\partial^{2}w}{\partial x^{2}}]$$

We introduce some dependencies $\sigma_{i}\left(e_{i}\right)$, which are used in this work. Analytical diagram for aluminum [43]:

(10) $$\sigma_{i}=\sigma_{s}\left[1-exp\left(-\frac{e_{i}}{e_{s}}\right)\right]$$

The second type of approximation for ideal elastic-plastic Prandtl body [44]:

(11) $$\sigma_{i}=3G_{0}e_{i}\;\mathrm{for}\;e_{i}<e_{s},\;\sigma_{i}=\sigma_{s}\;\mathrm{for}\;e_{i}\geq e_{s}$$

5. The von Mises criteria of plasticity is used;
6. For modeling size-dependent factors the modified couple stress theory [16,17] is applied. The governing equations of motion can be derived using Hamilton’s principle which can be stated as

(12) $$\overset{t_{1}}{\underset{t_{0}}{\int }}\left(\delta K-\delta U+\delta W_{q}+\delta W_{\varepsilon }\right)dt=0$$

where δK, δU and $\delta W_{q}$, $\delta W_{\varepsilon }$ denote virtual kinetic energy, virtual energy elastic deformation and virtual work done by external forces, respectively. The kinetic energy is

(13) $$K=\frac{1}{2}\overset{a}{\underset{0}{\int }}\overset{\frac{h}{2}}{\underset{-\frac{h}{2}}{\int }}\rho \left(x,\;z,\;e_{0},e_{i},t\right)\left\{{\left(\frac{\partial u}{\partial t}\right)}^{2}+{\left(\frac{\partial w}{\partial t}\right)}^{2}\right\}dzdx=\frac{1}{2}\overset{a}{\underset{0}{\int }}D_{1}\left\{{\left(\frac{\partial u}{\partial t}\right)}^{2}+{\left(\frac{\partial w}{\partial t}\right)}^{2}\right\}dx$$

where $D_{1}\left(x,t\right)=\int_{-\frac{h}{2}}^{\frac{h}{2}}\rho \left(x,z,e_{0},e_{i},t\right)dz$.

According to modified momentous theory [16,17], the deformation energy *U* taking into account small deformation is written by

(14) $$U=\frac{1}{2}\overset{a}{\underset{0}{\int }}\overset{\frac{h}{2}}{\underset{-\frac{h}{2}}{\int }}\left(\sigma_{xx}\epsilon_{xx}+2m_{xy}\chi_{xy}\right)dzdx$$

where $\varepsilon_{xx}$ and $\sigma_{xx}$ denote the components of the strain tensor and stress tensor respectively, $\chi_{xy}$—symmetric part of the curvature tensor, $m_{xy}$—deviatoric part of the couple stress tensor. The components are defined as follows

(15) $$\varepsilon_{xx}=\frac{\partial u}{\partial x}+\frac{1}{2}{\left(\frac{\partial w}{\partial x}\right)}^{2},\;\;\sigma_{xx}=\left(\lambda \left(x,z,e_{0},e_{i},t\right)+2\mu \left(x,z,e_{0},e_{i},t\right)\right)\varepsilon_{xx},$$

(16) $$\chi_{xy}=-\frac{1}{2}\frac{\partial^{2}w}{\partial x^{2}},\;\;m_{xy}=l^{2}\mu \chi_{xy},$$

in which, $\lambda \left(x,z,e_{0},e_{i},t\right),$ $\mu \left(x,z,e_{0},e_{i},t\right)$ are the Lamé constants, l is a material length scale parameter.

Substituting Equations (2)–(4), (15) and (16) into Equation (14), the strain energy of the functionally graded porous Euler–Bernoulli nanoscaled beam is expressed as

(17) $$U=\overset{a}{\underset{0}{\int }}\overset{\frac{h}{2}}{\underset{-\frac{h}{2}}{\int }}\frac{1}{2}\left\{\sigma_{xx}\left(\frac{\partial u}{\partial x}+\frac{1}{2}{\left(\frac{\partial w}{\partial x}\right)}^{2}\right)-\sigma_{xx}z\frac{\partial^{2}w}{\partial x^{2}}+2m_{xy}\left(-\frac{1}{2}\frac{\partial^{2}w}{\partial x^{2}}\right)\right\}dzdx=\;=\overset{a}{\underset{0}{\int }}\frac{1}{2}\left\{N_{x}\left(\frac{\partial u}{\partial x}+\frac{1}{2}{\left(\frac{\partial w}{\partial x}\right)}^{2}\right)-M_{x}\frac{\partial^{2}w}{\partial x^{2}}+2M_{x}^{\prime }\left(-\frac{1}{2}\frac{\partial^{2}w}{\partial x^{2}}\right)\right\}dx,$$

where $N_{x}$, $M_{x}$ are the classical resultant force and moment tractions and $M_{x}^{\prime }$—the couple stress moment of the beam. They are defined as:

(18) $$N_{x}=\overset{\frac{h}{2}}{\underset{-\frac{h}{2}}{\int }}\left(\lambda \left(x,z,e_{0},e_{i},t\right)+2\mu \left(x,z,e_{0},e_{i},t\right)\right)\left(\frac{\partial u}{\partial x}+\frac{1}{2}{\left(\frac{\partial w}{\partial x}\right)}^{2}\right)dz-\;-\overset{\frac{h}{2}}{\underset{-\frac{h}{2}}{\int }}\left(\lambda \left(x,z,e_{0},e_{i},t\right)+2\mu \left(x,z,e_{0},e_{i},t\right)\right)z\frac{\partial^{2}w}{\partial x^{2}}dz=C_{0}\left(\frac{\partial u}{\partial x}+\frac{1}{2}{\left(\frac{\partial w}{\partial x}\right)}^{2}\right)-C_{1}\frac{\partial^{2}w}{\partial x^{2}},\;M_{x}=\overset{\frac{h}{2}}{\underset{-\frac{h}{2}}{\int }}\left(\lambda \left(x,z,e_{0},e_{i},t\right)+2\mu \left(x,z,e_{0},e_{i},t\right)\right)z\left(\frac{\partial u}{\partial x}+\frac{1}{2}{\left(\frac{\partial w}{\partial x}\right)}^{2}\right)dz-\;-\overset{\frac{h}{2}}{\underset{-\frac{h}{2}}{\int }}\left(\lambda \left(x,z,e_{0},e_{i},t\right)+2\mu \left(x,z,e_{0},e_{i},t\right)\right)z^{2}\frac{\partial^{2}w}{\partial x^{2}}dz=C_{1}\left(\frac{\partial u}{\partial x}+\frac{1}{2}{\left(\frac{\partial w}{\partial x}\right)}^{2}\right)-C_{2}\frac{\partial^{2}w}{\partial x^{2}},\;M_{x}^{\prime }=\overset{\frac{h}{2}}{\underset{-\frac{h}{2}}{\int }}\mu \left(x,z,e_{0},e_{i},t\right)l^{2}\frac{\partial^{2}w}{\partial x^{2}}dz=D_{0}l^{2}\frac{\partial^{2}w}{\partial x^{2}},$$

where

(19) $$C_{d}\left(x,t\right)=\overset{\frac{h}{2}}{\underset{-\frac{h}{2}}{\int }}\left(\frac{E\left(x,z,e_{0},e_{i},t\right)\nu \left(x,z,e_{0},e_{i},t\right)}{\left(1+\nu \left(x,z,e_{0},e_{i},t\right)\right)\left(1-2\nu \left(x,z,e_{0},e_{i},t\right)\right)}+\frac{E\left(x,z,e_{0},e_{i},t\right)}{\left(1+\nu \left(x,z,e_{0},e_{i},t\right)\right)}\right)z^{d}dz,\;D_{0}\left(x,t\right)=\overset{\frac{h}{2}}{\underset{-\frac{h}{2}}{\int }}\frac{E\left(x,z,e_{o},e_{i},t\right)}{2\left(1+\nu \left(x,z,e_{0},e_{i},t\right)\right)}dz,\;\;\;\;D_{1}\left(x,t\right)=\overset{\frac{h}{2}}{\underset{-\frac{h}{2}}{\int }}\rho \left(x,z,e_{0},e_{i},t\right)dz,\;\;\;\;d=0,1,2.$$

Variation of work is done by external loads has the form:

(20) $$\frac{\partial }{\partial x}\left(C_{0}(x,t)\left(\frac{\partial u}{\partial x}+\frac{1}{2}{\left(\frac{\partial w}{\partial x}\right)}^{2}\right)-C_{1}(x,t)\frac{\partial^{2}w}{\partial x^{2}}\right)=D_{1}\frac{\partial^{2}u}{\partial t^{2}},\;\frac{\partial^{2}}{\partial x^{2}}\left(C_{1}(x,t)\left(\frac{\partial u}{\partial x}+\frac{1}{2}{\left(\frac{\partial w}{\partial x}\right)}^{2}\right)-\left(C_{2}(x,t)+D_{0}(x,t)l^{2}\right)\frac{\partial^{2}w}{\partial x^{2}}\right)+\;+\frac{\partial }{\partial x}\left(C_{0}(x,t)\left(\frac{\partial u}{\partial x}+\frac{1}{2}{\left(\frac{\partial w}{\partial x}\right)}^{2}\right)-C_{1}(x,t)\frac{\partial^{2}w}{\partial x^{2}}\right)+q=D_{1}\frac{\partial^{2}w}{\partial t^{2}}+\varepsilon D_{1}\frac{\partial w}{\partial t},$$

where: *t*—time, $q=q_{0}+q_{1}\left(x,t\right)$—uniformly distributed transverse load, *ε*—damping coefficient of the surrounding matter, l is a material length scale parameter, *ρ*—density of a material. The law of load change in time and along the axis of the beam can be arbitrary. One of the boundary conditions and initial conditions, which corresponds to Equation (20) are expressed by

1. Fixed support (both ends x=0, x=a are fixed):
  (21) $$w(0,t)=w(a,t)=\frac{\partial w(0,t)}{\partial x}=\frac{\partial w(a,t)}{\partial x}=u(0,t)=u(a,t)=0,$$
2. pinned support (both ends x=0, x=a are pinned):
  (22) $$w(0,t)=w(a,t)=\frac{\partial^{2}w(0,t)}{\partial x^{2}}=\frac{\partial^{2}w(a,t)}{\partial x^{2}}=u(0,t)=u(a,t)=0,$$
3. one beam end is fixed, x=0 whereas the second one is pinned x=a:
  (23) $$w(0,t)=w(a,t)=\frac{\partial w(0,t)}{\partial x}=\frac{\partial^{2}w(a,t)}{\partial x^{2}}=u(0,t)=u(a,t)=0.$$

The following initial conditions are applied:

(24) $$w(x,0)=0,u(x,0)=0,\frac{\partial w(x,0)}{\partial t}=0,\frac{\partial u(x,0)}{\partial t}=0.$$

To carry out the numerical analysis, the following dimensionless parameters are introduced:

$$\bar{w}=\frac{w}{h},\;\;\bar{u}=\frac{ua}{h_{}^{2}},\;\;\bar{x}=\frac{x}{a},\;\;\bar{z}=\frac{z}{h},\lambda_{1}=\frac{a}{h},\;\;\bar{q}=q\frac{a^{4}}{h_{}^{4}E_{0}},\gamma =\frac{l}{h},\bar{t}=\frac{t}{\tau },\;\;\tau =\frac{a}{c},\;\;c=\sqrt{\frac{E_{0}}{\rho_{0}}},\;\;\;\bar{\varepsilon }=\varepsilon \frac{a}{c},\bar{\rho }\left(x,z,e_{0},e_{i},t\right)=\frac{\rho \left(x,z,e_{0},e_{i},t\right)}{\rho_{0}},\;\bar{E}\left(x,z,e_{0},e_{i},t\right)=\frac{E\left(x,z,e_{0},e_{i},t\right)}{E_{0}},$$

where γ—the size dependent parameter, $E_{0}$ and $\rho_{0}$—the Young’s modulus and density of ceramics.

Three types of distributions of porosity across the thickness of the material (2)–(4) for physically and geometrically nonlinear PFG nanoscaled Euler–Bernoulli beams (Appendix A, (A1)–(A4)) were applied:

1. Uniform porosity (U-PFGM):

(25) $$\varphi \left(z\right)={\left(\frac{1}{2}+\frac{z}{h}\right)}^{k},\;\psi \left(z\right)=1;$$

2. Reduced porosity from the top and bottom surfaces to the center (X-PFGM)

(26) $$\varphi \left(z\right)={\left(\frac{1}{2}+\frac{z}{h}\right)}^{k},\;\psi \left(z\right)=\left(\frac{1}{2}-\frac{\left[z\right]}{h}\right);$$

3. Increased porosity at the top and bottom the surfaces (O-PFGM)

(27) $$\varphi \left(z\right)={\left(\frac{1}{2}+\frac{z}{h}\right)}^{k},\;\psi \left(z\right)=\frac{\left[z\right]}{h};$$

where *k*—represents the graded index of material property.

## 3. Solution Methods

PDEs system taking into account both geometric and physical non-linearities of PFG nanoscaled Euler–Bernoulli beam (Appendix A, (A1)–(A4)), as well as taking into account only the physical nonlinearity (PN) (Appendix A, (A6)–(A10)) or the geometric nonlinearity (GN) (Appendix A, (A11)–(A15)) has a complex structure. An analytical solution of systems of PDEs (Appendix A, (A1)–(A4)) is not possible. We searched for the solution numerically. In this paper, we propose a new methodology for solving complex systems of PDEs. Partial Derivative Equations (PDEs) using the second-order accuracy finite difference method (FDM) on spatial coordinates reduces to a system of ordinary differential equations (ODEs), i.e., to the Cauchy problem. The rationale for the use of the finite difference method for the study of beam structures is given on the basis of computation Germaine Lagrange plate, taking into account the modified couple stress theory [16] under the action of a constant static load. Computation performed by the Finite Element Method (Triangular Elements and Quadrangular Elements) and Finite Difference Method are given in [45]. To obtain an exact solution, the number of partitions for Triangular Elements—350, for Quadrangular Elements—165 and for the Finite Difference Method 35 × 35 = 1225 was used. For the same equation computation were performed by the methods presented in [45], namely Kantorovich–Vlasov Methods [46,47], Variational Iteration Method [48,49], Vaindiner’s method [50], Bubnov–Galerkin Method [51], and Navier method. Awrejcewicz et al. [45] showed the high efficiency of the Finite Difference Method for plates. For beams described by functionally graded porous physically nonlinear equations investigated in this article, the efficiency of the Finite Difference Method is high. Next, to obtain reliable results the system of ODEs by several methods, namely the Runge–Kutta methods from the 4th to the 8th order of accuracy is solved [52,53,54,55]. The convergence of the finite difference method depending on the partitioning of the interval $x\in \left[0;1\right]$ in the spatial coordinate and in time is studied. It is necessary to fulfill the condition coincidence of the main functions *w*(*x*, *t*), *u*(*x*, *t*) and their derivatives up to the second order, inclusive, depending on the number of partitions in the finite difference method on spatial coordinates and time. In this way, the problem as a system with an “almost” infinite number of degrees of freedom is considered. Efficiency showed a spatial grid $x\in \left[0;1\right]$, $z\in \left[-1/2;\;1/2\right]$ with partitions *nx* = 100, *nz* = 14. Note that at each time step, the iterative procedure is constructed by the Birger’s method of variable elasticity parameters [40]. Vorovich et al. [56] submitted proof of the Birger’s method of variable elasticity parameters. Krysko et al. [57] proved the existence of this class of problems. In this way, the proposed method is correct, because each step has a rigorous mathematical proof [56]. This established a method or method continuation of the solution by parameter. According to this method, the dissipation coefficient ε is considered as known and the dynamic problem will be solved for the load $q_{0}=const$. Krysko et al. [58] showed the effectiveness of this method.

## 4. Numeric Experiments and the Results Discussion

In this section, porous functionally graded nanoscaled Euler–Bernoulli beams to validate the effectiveness and accuracy of the proposed new mathematical model are studied. In the following examples, numerical studies for beam structures considering three types of non-linearities: physical nonlinearity (FN), geometric nonlinearity (GN) and both physical and geometric (FGN) nonlinearity are present.

This section may be divided by subheadings. It should provide a concise and precise description of the experimental results, their interpretation, as well as the experimental conclusions that can be drawn.

### 4.1. Statics of a Physically Nonlinear Porous Functionally Graded Nanoscaled Euler–Bernoulli Beam

Consider the beam introduced in Section 2. To study the physically nonlinear PFG nanoscaled Euler–Bernoulli beams, it is necessary to set the physical parameters of the FG material. The material parameters of the beam for metal (*Al*) and ceramics (*SiC*) are given by Table 1. 

**Figure 2:**
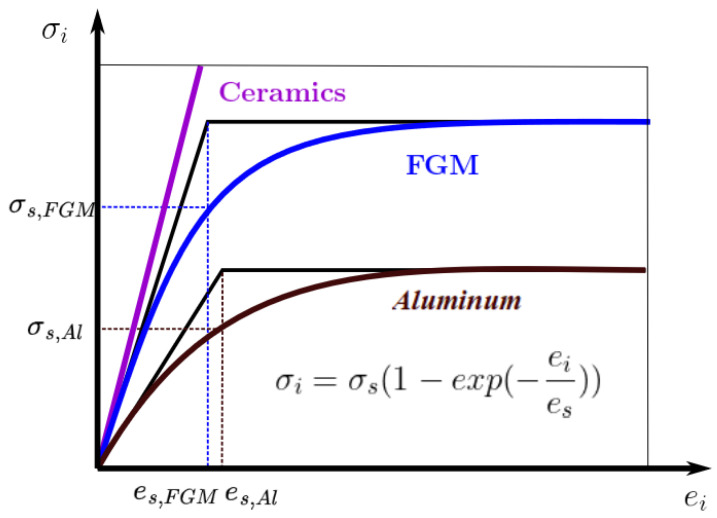
Dependence $\sigma_{i}\left(e_{i}\right)$ for metal (*Al*), ceramics (*SiC*) and functionally graded material (FGM).

Recall, as we assumed in Section 2 for our mathematical model, the Young’s modulus $E_{m}$, the Poisson’s ratio *ν_m_* and density of the beam material *ρ_m_* are nonlinear and depend both on the coordinates *x*, *z* and on the stress–strain state *e_i_*. For this case, the analytical diagram for aluminum by (10). Figure 2 shows graphical and analytical $\sigma_{s}\left(e_{i}\right)$ dependence. For aluminum colored brown (first type of approximation (10)) and black (second type of approximation (11)), for ceramics, the physical parameters assumed constant and colored in purple (first type of approximation (10)) and for the FGM in blue (first type of approximation (10) and black (second type of approximation (11)) [38]. 

In order to evaluate stress–strain state consider PFG nanoscaled Euler–Bernoulli beam (Appendix A, (A6), (A7) and (A10)) taking into account FN. 

At each time step, an iterative procedure of the method of Birger’s variable elasticity parameters is applied [40]. This procedure was introduced in [57,59] for the calculation of FN metal plates in a three-dimensional formulation by the finite element method.

Let us apply the establishment method [58] to the solution of equations. Number of partitioning meshes are: nx=100, nz=14.

**Figure 3:**
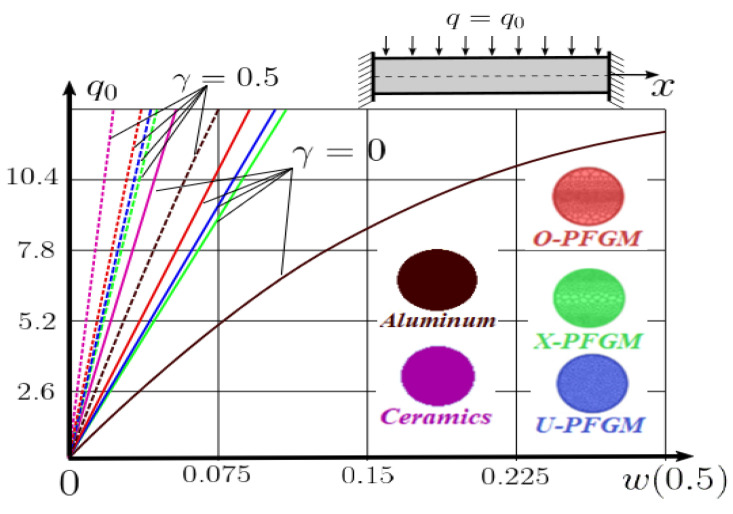
The value of the load—deflection $q_{0}\left(w\left(0.5\right)\right)$ at *λ*_1_ = 30 physically nonlinear PFG nanoscaled beam versus the size-dependent parameter γ.

**Figure 4:**
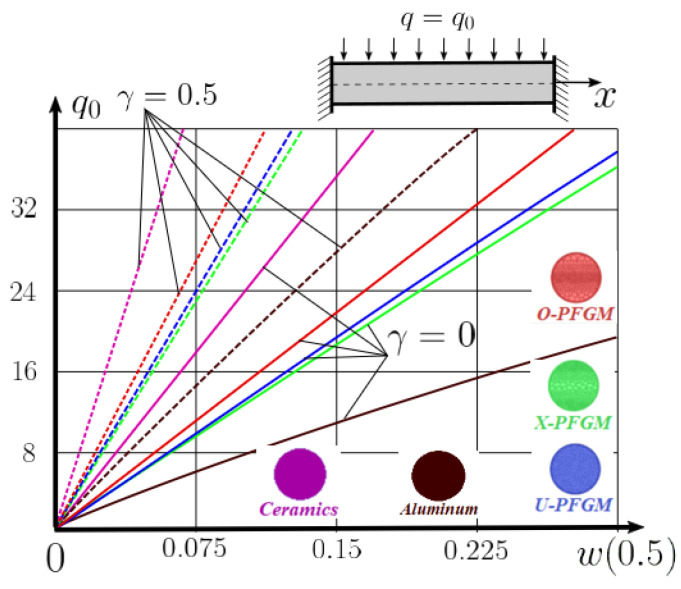
The value of the load—deflection $q_{0}\left(w\left(0.5\right)\right)$ at *λ*_1_ = 50 physically nonlinear PFG nanoscaled beam versus the size-dependent parameter γ.

**Figure 5:**
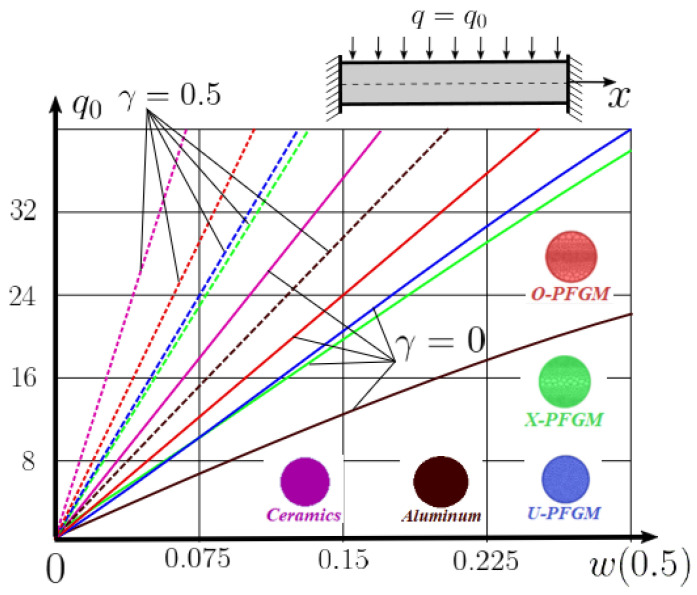
The value of the load—deflection $q_{0}\left(w\left(0.5\right)\right)$ at *λ*_1_ = 100 physically nonlinear PFG nanoscaled beam versus the size-dependent parameter γ.

**Figure 6:**
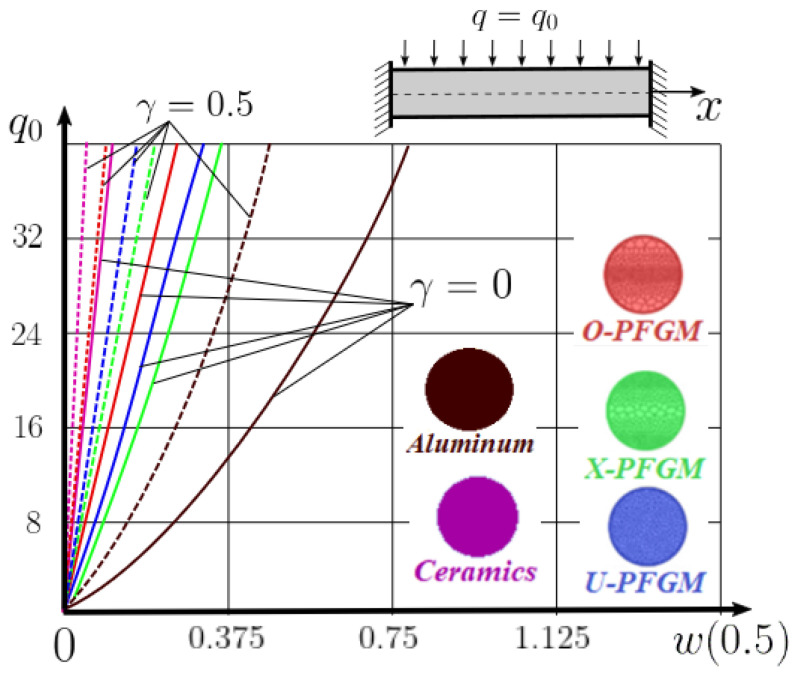
The value of the load—deflection $q_{0}\left(w\left(0.5\right)\right)$ at *λ*_1_ = 30 geometrically PFG nanoscaled beam versus the size-dependent parameter γ.

The “load-deflection” dependences in the center of the beam $q_{0}\left(w\left(0.5\right)\right)$ for some values of the dimensional parameter γ=0;0.5 are presented in Figure 3, Figure 4, Figure 5 and Figure 6. The nanoscale is not taken into account at γ=0 and taken into account in case γ=0.5 The porosity functionally graded nanoscaled beams material are described by various models (25–27) taking into account the power coefficient *k* = 1 and the porosity index Γ = 0.4, as well as ceramics (*SiC*) and pure aluminum (10).

From the analysis of the results presented on Figure 3, Figure 4, Figure 5 and Figure 6 regarding the static behavior of the physically nonlinear (FN) of nanobeam, we can conclude that for any ratio of length to thickness *a/h*, the bearing capacity for a ceramic beam exceeds that for a beam made from PFGM or aluminum. However, the bearing capacity of a beam with porosity distributions modeled by various power functions (U-PFGM, X-PFGM, O-PFGM) is higher than for an aluminum beam. The bearing capacity of the beam with the porosity distributions O-PFGM is higher than for beam with porosity distributions U-PFGM and X-PFGM. Deflections of beams with the porosity distributions U-PFGM and X-PFGM are almost the same. Analysis of the results shows a significant influence of the size-dependent parameter on the stress–strain state of the nanobeam. Table 2, Table 3, Table 4 and Table 5 respectively show diagrams deflection *w(x)*, $x\in \left[0;0.5\right]$ and their second derivatives $w^{\prime \prime }\left(x\right),\;x\in \left[0.5;1\right]$. In view of the symmetry of the boundary conditions (34), the zones of plastic and elastic deformations are colored orange and gray, respectively. The employed data are: *a/h* = 30, *γ* = 0 (Table 2), *a/h* = 30, *γ* = 0.5 (Table 3), *a/h* = 50, *γ* = 0 (Table 4), *a/h* = 100, *γ* = 0 (Table 5).

Based on the results reported in Table 2, Table 3, Table 4 and Table 5 respectively, we can conclude that, for an aluminum beam, taking into account physical non-linearity and boundary conditions, fixed support with a ratio of length to thickness of the plastic zone for the first time appear in the corners, and at increasing load—in the center of the beam. Without taking into account the physical non-linearity, zones of the plasticity are located symmetrical about beam thickness. Deflection *w*(*x*) and their second derivatives *w*″(*x*) of the aluminum beams significantly more than for beam with the porosity distributions U-PFGM, X-PFGM and O-PFGM. Accounting the porosity distributions for the functionally graded nanoscaled Euler–Bernoulli beams leads to a decrease in plastic deformation zones and taking into account the size-dependent parameter *γ* (*γ* = 0.5) leads to a reduction of elastic-plastic deformations, and in some cases to their disappearance (*λ*_1_ = 50, *λ*_1_= 100, Table 4 and Table 5).

### 4.2. Statics of a Geometrically Nonlinear Porous Functional Graded Nanoscaled Euler–Bernoulli Beam

Now consider the static solution of a geometrically nonlinear PFG nanoscaled Euler–Bernoulli beam rigidly clamped on the edges (Appendix A, (A12)) under the action of a constant transverse load q_0_. Solved geometrically nonlinear PDEs (Appendix A, (A11), (A12), (A15)) by the establishment method with a dissipation coefficient ε = 3, but without using the Birger method of variable elasticity parameters. The method of finite differences of the second order of accuracy for *x*∈[0; 1], *nx* = 100, *λ*_1_ = 30 is applied. The material length scale parameter of beam is assumed to be γ=0;0.5. The porosity distributions of the beam are taken into account by approximations U-PFGM (25), X-PFGM (26), O-PFGM (27). Figure 6 are illustrates the calculation results. The color scheme of the curves is the same as in the previous paragraph 4.1. Solid curves for γ=0, dashed for γ=0.5.

Analysis of the results shows a significant influence of the size-dependent parameter γ on the bearing capacity of the geometrically nonlinear porous functionally graded nanoscaled Euler–Bernoulli beam. Load-bearing capacity of a beam, taking into account any porosity distributions significantly higher for γ=0.5, than for γ=0. At the same time, the bearing capacity of the beam with porosity distributions O-PFGM (27) is higher than with U-PFGM (25) and X-PFGM (26).

### 4.3. Statics of a Physically and Geometrically Nonlinear Porous Functional Graded Nanoscaled Euler–Bernoulli Beam 

Recall that in Section 2 of the paper, a new mathematical model of statics and dynamics of porous functionally graded (PFG) Euler–Bernoulli nanoscaled beam, taking into account the physical and geometric of nonlinearities (FGN) was constructed. In addition, the statics of a physically or geometrically nonlinear porous functional graded nanoscaled Euler–Bernoulli beam have been studied depending on the values of the size-dependent parameter, the load and the size of the beam. Three types of porosity distributions by the power function [U-PFGM (25), X-PFGM (26), O-PFGM (27)] were taken into account for material modeling.

Let us present the results of a numerical study of the statics of both a physically and geometrically nonlinear porous functional graded (FGNPFG) nanoscaled beam. The establishment method [58] with a dissipation coefficient *ε* = 3 is applied. The solution method and algorithm in Section 4.1 were described. The finite difference method of the second order of accuracy was used to reduce a partial differential equation to the Cauchy problem. Numerical results were obtained by solving the Cauchy problem by the Runge–Kutta method from the fourth to the eighth order of accuracy and by the Newmark method. As in Section 4.1 is built from an iterative procedure the Birger method of variable elasticity parameters at each time. Number of partitioning meshes are by length *x*∈[0; 1], *nx* = 100 and on thickness *z*∈[−1/2; 1/2], *nz* = 14. At solving problems, plasticity zones according to Von Mises criteria of plasticity were built. The deformation diagram was taken into account by formula (10). The stress–strain state of PFG nanoscaled Euler–Bernoulli beams was studied depending on the magnitude of the load $q_{0}$, i.e., dependence $q_{0}\left(w\left(0.5\right)\right)$ of the material length scale parameter *γ* = 0; 0.5, types of porosity distributions (25–27) and parameter *λ*_1_ = 30; 50; 100. Note that the convergence of the developed method requires the coincidence of functions and their derivatives up to the second order inclusive. In our case, problems were studied as systems with an “almost” infinite number of degrees of freedom. The requirement that the coincidence of *w*(*x*) up to and including the second derivatives *w*″(*x*) for *x*∈[0; 1] indicates that an almost exact solution was obtained using the finite difference method with a partition of *nx* = 100, *nz* = 14. 

**Figure 7:**
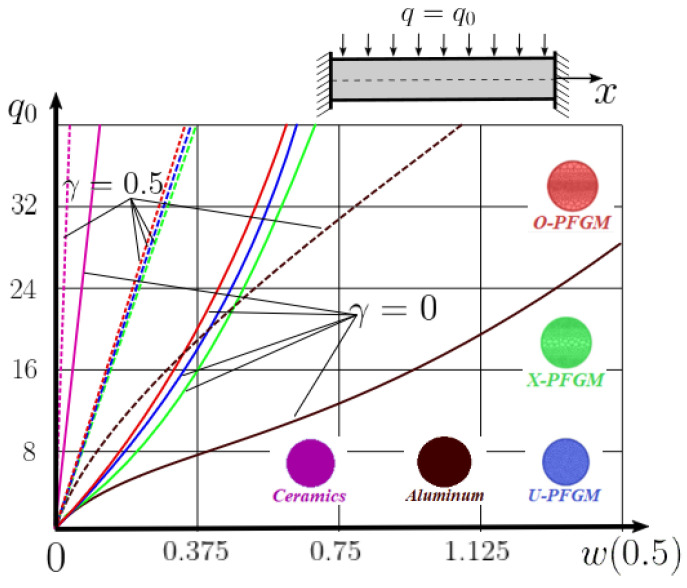
The value of the load—deflection $q_{0}\left(w\left(0.5\right)\right)$ at *λ*_1_ = 30 FGNPFG nanoscaled beam versus the size-dependent parameter *γ*.

**Figure 8:**
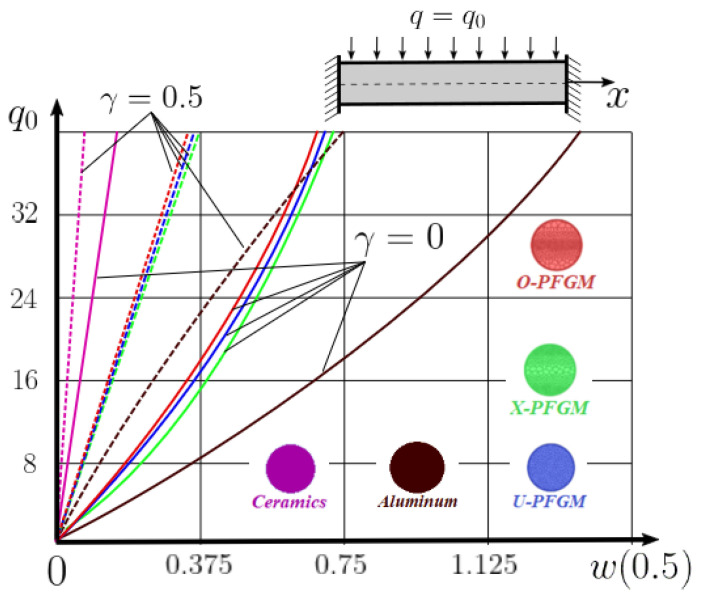
The value of the load—deflection $q_{0}\left(w\left(0.5\right)\right)$ at *λ*_1_ = 50 FGNPFG nanoscaled beam versus the size-dependent parameter *γ*.

**Figure 9:**
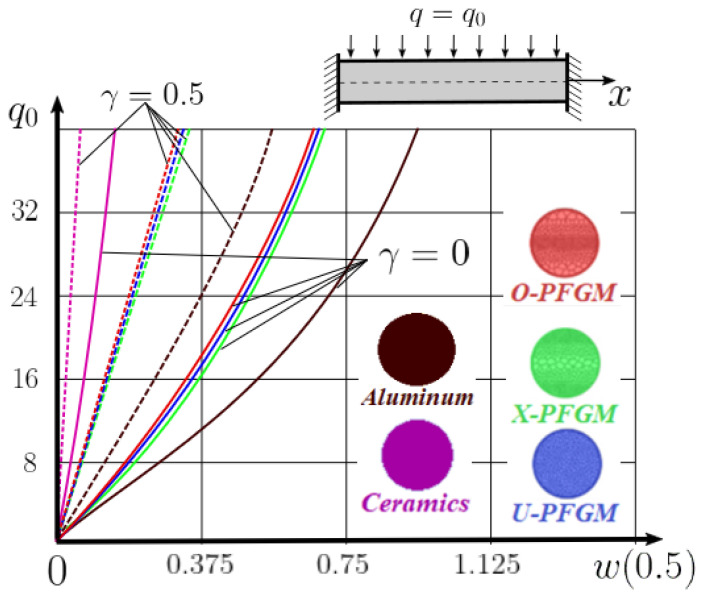
The value of the load—deflection $q_{0}\left(w\left(0.5\right)\right)$ at *λ*_1_ = 100 FGNPFG porous nanoscaled beam versus the size-dependent parameter *γ*.

Calculation results of elastic-plastic porous functionally graded nanoscaled Euler–Bernoulli beams according to the method described above are shown in Figure 7, Figure 8 and Figure 9.

The dependencies “load-deflection” ($q_{0}\left(w\left(0.5\right)\right)$) for the center of the beam for some values of the material length scale parameter γ = 0; 0.5 are represented graphically (Figure 7, Figure 8 and Figure 9).

Table 6, Table 7, Table 8, Table 9 and Table 10 show the zones of elastic-plastic deformations (plastic deformations—orange color, elastic deformations—gray color), as well as deflection diagrams *w(x)*, *x*∈[0; 0.5] and diagrams of second derivatives *w*″*(x), x*∈[0.5; 1] for the corresponding parameters.

Table 6, Table 7, Table 8, Table 9 and Table 10 show the non-symmetry of the distribution of plastic deformations over the thickness for the considered materials. At studying the stress–strain state of an aluminum beam by taking into account both geometric and physical nonlinearity (elastic-plastic deformation according to the deformation theory of plasticity), the phenomenon of the appearance of a plastic hinge was discovered for the first time for beams with fixed support boundary conditions. This leads to a change in the estimated beam scheme during loading and to a change in the boundary conditions from fixed support to pinned support. In this case, deflection *w(x)* $\mathrm{at}\;x\in \left[0;0.5\right]$ and their second derivatives *w*″*(x)* at $x\in \left[0.5;1\right]$ change in the same way. Note that we observe a new type of nonlinearity, which is called “constructive” non-linearity. In the case of constructive nonlinearity, the boundary conditions change as a result of deformation.

Table 11 and Table 12 show the values of the beam deflection $w\left(0.5\right)$ in the center, taking into account both the geometric and physical nonlinearities (Table 11) and taking into account only the geometric nonlinearity (Table 12) with porosity distributions U-PFGM, X-PFGM, O- PFGM for *λ*_1_ = 30; 50; 100, γ=0;0.5 at load *q*_0_ = 8.

From the above results, it is known, that the bearing capacity of a beam taking into account GN and elastic-plastic deformations according to the deformation theory of plasticity (Figure 7) is less than taking into account only the geometric nonlinearity (Figure 6). A detailed analysis of the deflections in the center of the beam *w*(0.5) for different loads (Table 11 and Table 12) shows that the deflection of a beam at *λ*_1_ = 30 made of aluminum, taking into account two types of nonlinearities, differs from the deflection, taking into account only one type of nonlinearity, almost by two times or 100%. An interesting fact is that if parameter *λ*_1_ = 50; 100, then the effect of impact of physical nonlinearity becomes much smaller and will be approximately 15–20%. In addition, the physical nonlinearity must be taken into account upon calculation of the nanoscaled aluminum beams (γ=0.5) because in this case the deflection differs by 20–50%.

We obtained similar results when constructing a design scheme for a PFG beams. However, the mass fraction of aluminum in the porous structure is less, so the effect of physical nonlinearity is less compared to a pure aluminum beam, but is still significant and varies from 5% to 55% depending on the values of the parameters *λ*_1_ and γ. For beams, taking into account two types of nonlinearities, the numerical results are the most accurate. This allows you to take into account the real work of the beams.

Influence of different values of the power parameter *k* = 0.2; 0.5; 1 was studied. The power parameter defines the ratio of material volume fractions, on the static behavior of macroscaled and nanoscaled FGNPFG beams (Table 13). At increases in *k*, the volume fraction of aluminum also increases. For any type of boundary conditions, the decrease in parameter k leads to an increase in bearing capacity of the beam. The type of boundary conditions significantly affects the stress–strain state. For a fixed support beam (Appendix A, (A2)) the influence of the porosity type is not significant compared to a pinned support beam (Appendix A, (A3)).

The effect of the porosity index Γ (no porosity at Γ = 0) on the static behavior of a pinned support geometrically nonlinear porous functional graded (FGNPFG) beam (Appendix A, (A3)) (Table 14) was studied. The power coefficient was *k* = 1. With an increase in material porosity, the bearing capacity of the beam decreases slightly for the O-PFGM material. The porosity index (G) significantly affects the stress–strain state of a beam made of U-PFGM and X-PFGM materials.

## 5. Conclusions

In the present work, a new mathematical model of PFG nanoscaled Euler–Bernoulli beam taking into account elasto-plastic deformations on the deformation theory of plasticity (FN) and geometric nonlinearity (GN) in the form of Von Kármán is presented. The Hamilton’s principle is applied to derive the governing equations of the beam as well as boundary and initial conditions.

1. The developed methodology for calculating elastic-plastic flexible PFG nanoscaled Euler–Bernoulli beams allows us to consider them as a system with an “almost” infinite number of degrees of freedom;
2. The calculations of PFG elastic-plastic nanoscaled Euler–Bernoulli beams according to the deformation theory of plasticity are performed by accounting only for active loading (no unloading) at each time step;
3. Upon studying the stress–strain state of an aluminum beam taking into account both geometric and physical nonlinearity (FGN), the phenomenon of the appearance of a plastic hinge was discovered for the first time. The new type of nonlinearity is called “constructive” non-linearity (CN);
4. In the case of CN, the boundary conditions change due to deformation;
5. The numerical results reveal that accounting for the size-dependent parameter leads both to a significant change in the stress–strain state and to a reduction in zones of the plastic deformation of the nanobeam;
6. The bearing capacity of a PFG beam increases depending on the parameters γ and *λ*_1_;
7. The stress–strain state of physically and geometrically nonlinear beams under the action of a uniformly distributed transverse load is significantly affected by boundary conditions. The type of porosity distribution with a change the parameters γ, *k*, Γ affect slightly on the stress–strain state of the pinned support beam (Appendix A, (A3)) and significantly effect the stress–strain state for a fixed support (Appendix A, (A2)) beam.

The mathematical model and calculation methodology presented in this paper can be used for other size-dependent theories. Additionally, the proposed approach may be extended to different types of nanoscaled structures such as nanoscaled plates and shells.

## Figures and Tables

**Table 1:** The material parameters of the beam.

Symbol	Base Quantity	Base Unit	Symbol	Base Quantity	Base Unit
*ε*	dissipation coefficient	3	γ	the dimensional parameter	0; 0.5
$E_{m}$	modulus of elasticity of pure aluminum (*Al*)	70,000 MPa	$E_{c}$	modulus of elasticity of ceramics (*SiC*)	410,000 MPa
$\nu_{m}$	poisson’s ratio of pure aluminum (*Al*)	0.34	$\nu_{m}$	poisson’s ratio of ceramics (*SiC*)	0.19
$\rho_{m}$	density ofpure aluminum (*Al*)	2712 kg/m^3^	$\rho_{m}$	density of ceramics (*SiC*)	3100 kg/m^3^
$\sigma_{s}$	values of stress yield	102.3 MΠa	$e_{s}$	yield strain	0.98 × 10^−3^
*k*	the power coefficient	0.2; 0.5; 1	Γ	porosity index	0; 0.2; 0.4

**Table 2:** Distribution of plastic and elastic deformations along the length and thickness of the beam, the value of the deviation function *w(x)*, $x\in \left[0;0.5\right]$ and its second derivative $w^{\prime \prime }\left(x\right),\;x\in \left[0.5;1\right]$ versus the parameters: *λ*_1_ = 30, *γ* = 0, *q*_0_ = 12.

Material		The Value of the Deviation Function *w(x)*, $\;x\in \left[0;0.5\right]$ and Its Second Derivative $w^{\prime \prime }\left(x\right),\;x\in \left[0.5;1\right]$	
Metal (*Al*)		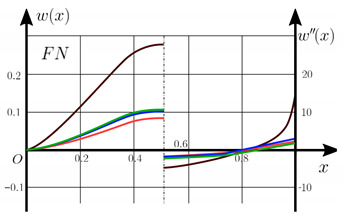	
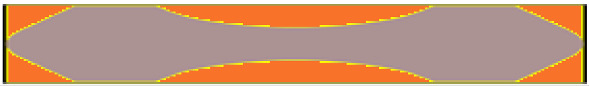			
U-PFGM			
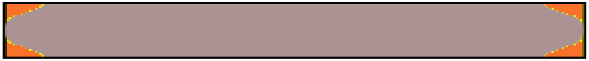			
X-PFGM			
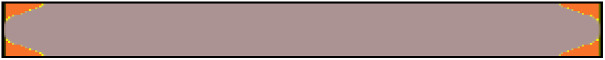			
O-PFGM			
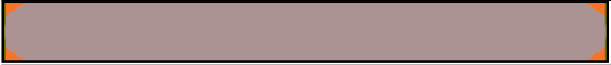			
Conditional designations			
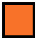	Plastic zones	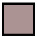	Elastic zones
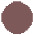	Metal (*Al*)	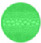	X-PFGM
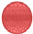	O-PFGM	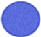	U-PFGM

**Table 3:** Distribution of plastic and elastic deformations along the length and thickness of the beam, the value of the deviation function *w(x)* at $x\in \left[0;0.5\right]$ and its second derivative $w^{\prime \prime }\left(x\right)\;\mathrm{at}\;x\in \left[0.5;1\right]$ versus the parameters: *λ*_1_ = 30, *γ* = 0.5, *q*_0_ = 12.

Material	The Value of the Deviation Function *w(x)*, $\;x\in \left[0;0.5\right]$ and Its Second Derivative $w^{\prime \prime }\left(x\right),\;x\in \left[0.5;1\right]$
Metal (*Al*)	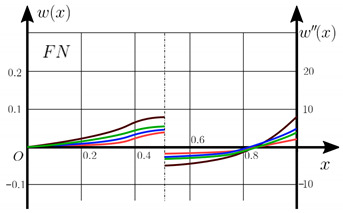
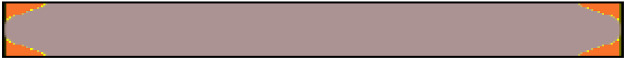	
U-PFGM	
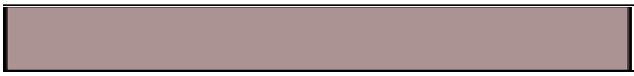	
X-PFGM	
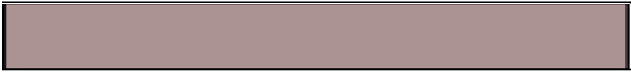	
O-PFGM	
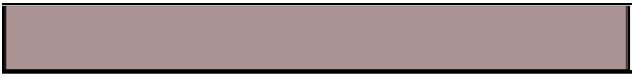	

**Table 4:** Distribution of plastic and elastic deformations along the length and thickness of the beam, the value of the deviation function *w(x)* at $x\in \left[0;0.5\right]$ and its second derivative $w^{\prime \prime }\left(x\right)\;\mathrm{at}\;x\in \left[0.5;1\right]$ versus the parameters: *λ*_1_ = 50, *γ* = 0, *q*_0_ = 19.

Material	The Value of the Deviation Function *w(x)*, $\;x\in \left[0;0.5\right]$ and Its Second Derivative $w^{\prime \prime }\left(x\right),\;x\in \left[0.5;1\right]$
Metal (*Al*)	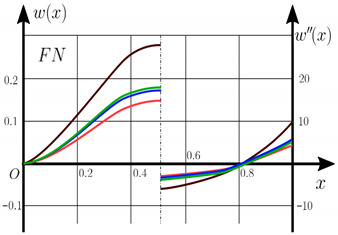
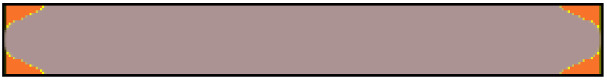	
U-PFGM	
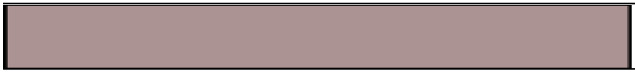	
X-PFGM	
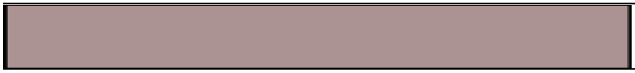	
O-PFGM	
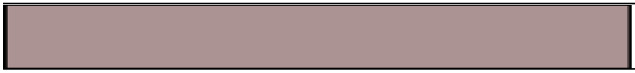	

**Table 5:** Distribution of plastic and elastic deformations along the length and thickness of the beam, the value of the deviation function *w(x)* at $x\in \left[0;0.5\right]$ and its second derivative $w^{\prime \prime }\left(x\right)\;\mathrm{at}\;x\in \left[0.5;1\right]$ versus the parameters: *λ*_1_ = 100, *γ* = 0, *q*_0_ = 22.

Material	The Value of the Deviation Function *w(x)*, $\;x\in \left[0;0.5\right]$ and Its Second Derivative $w^{\prime \prime }\left(x\right),\;x\in \left[0.5;1\right]$
Metal (*Al*)	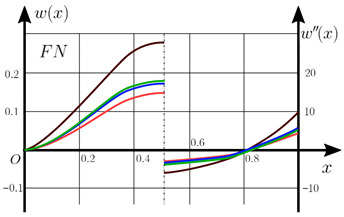
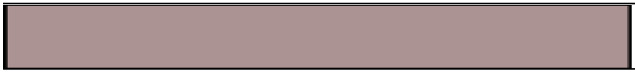	
U-PFGM	
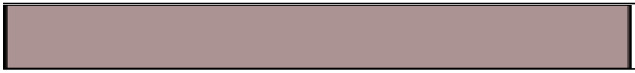	
X-PFGM	
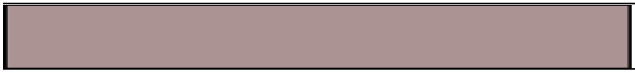	
O-PFGM	
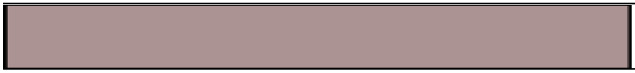	

**Table 6:** Distribution of plastic zones, the value of the deviation function $w\left(x\right)\;\mathrm{at}\;x\in \left[0;0.5\right]$ and its second derivative *w*″*(x)* at $x\in \left[0.5;1\right]$ versus the parameters: *λ*_1_ = 30, *γ* = 0, *q*_0_ = 45.

Material		The Value of the Deviation Function *w(x)*, $\;x\in \left[0;0.5\right]$ and Its Second Derivative $w^{\prime \prime }\left(x\right),\;x\in \left[0.5;1\right]$	
Metal (*Al*)		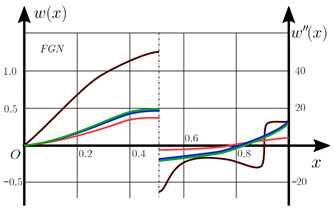	
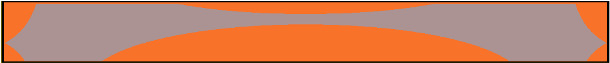			
U-PFGM			
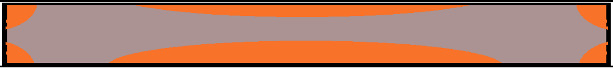			
X-PFGM			
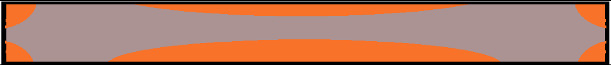			
O-PFGM			
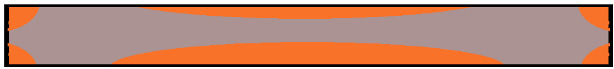			
Conditional designations			
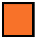	Plastic zones	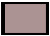	Elastic zones
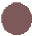	Metal (*Al*)	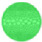	X-PFGM
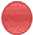	O-PFGM	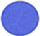	U-PFGM

**Table 7:** Distribution of plastic zones, the value of the deviation function $w\left(x\right),\;x\in \left[0;0.5\right]$ and its second derivative *w*″*(x)* at $x\in \left[0.5;1\right]$ versus the parameters: *λ*_1_ = 30, *γ* = 0.5, *q*_0_ = 45.

Material		The Value of the Deviation Function *w(x)*, $\;x\in \left[0;0.5\right]$ and Its Second Derivative $w^{\prime \prime }\left(x\right),\;x\in \left[0.5;1\right]$	
Metal (*Al*)		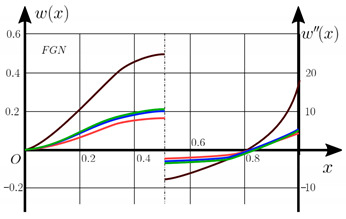	
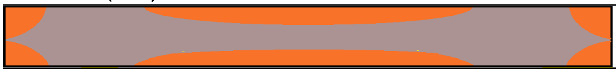			
U-PFGM			
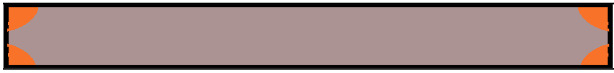			
X-PFGM			
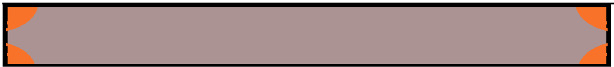			
O-PFGM			
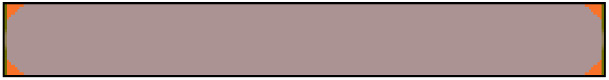			
Conditional designations			
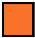	Plastic zones	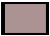	Elastic zones
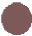	Metal (*Al*)	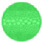	X-PFGM
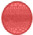	O-PFGM	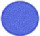	U-PFGM

**Table 8:** Distribution of plastic zones, the value of the deviation function $w\left(x\right)\;x\in \left[0;0.5\right]$ and its second derivative *w*″*(x)* at $x\in \left[0.5;1\right]$ versus the parameters: *λ*_1_ = 50, *γ* = 0, *q*_0_ = 100.

Material	The Value of the Deviation Function *w(x)*, $\;x\in \left[0;0.5\right]$ and Its Second Derivative $w^{\prime \prime }\left(x\right),\;x\in \left[0.5;1\right]$
Metal (*Al*)	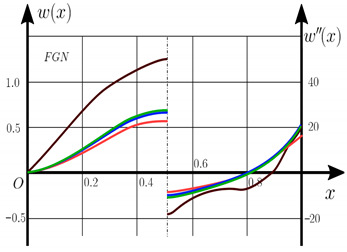
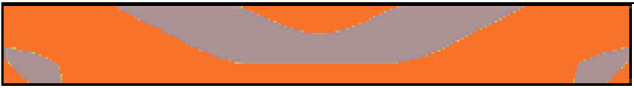	
U-PFGM	
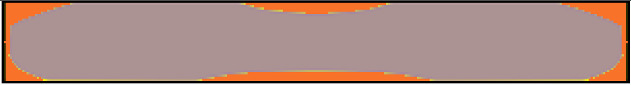	
X-PFGM	
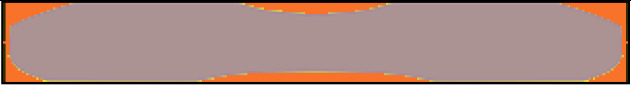	
O-PFGM	
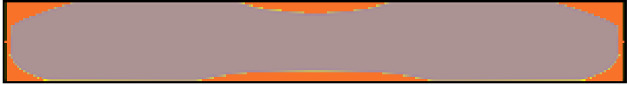	

**Table 9:** Distribution of plastic zones, the value of the deviation function $w\left(x\right)\;x\in \left[0;0.5\right]$ and its second derivative *w*″*(x)* at $x\in \left[0.5;1\right]$ versus the parameters: *λ*_1_ = 50, *γ* = 0.5, *q*_0_ = 100.

Material	The Value of the Deviation Function *w(x)*, $\;x\in \left[0;0.5\right]$ and Its Second Derivative $w^{\prime \prime }\left(x\right),\;x\in \left[0.5;1\right]$
Metal (*Al*)	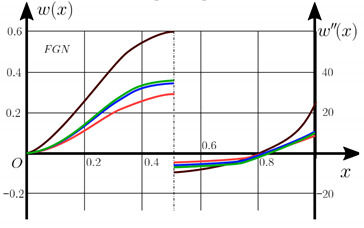
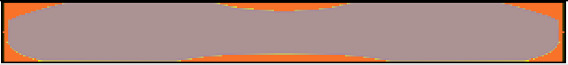	
U-PFGM	
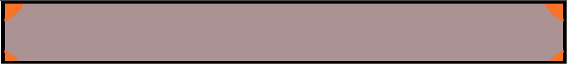	
X-PFGM	
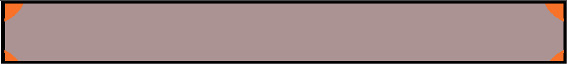	
O-PFGM	
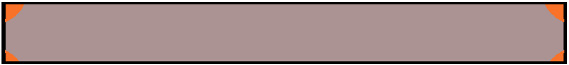	

**Table 10:** Distribution of plastic zones, the value of the deviation function $w\left(x\right)\;\mathrm{at}\;x\in \left[0;0.5\right]$ and its second derivative *w*″*(x)* at $x\in \left[0.5;1\right]$ versus the parameters: *λ*_1_ = 100, *γ* = 0, *q*_0_ = 100.

Material	The Value of the Deviation Function *w(x)*, $\;x\in \left[0;0.5\right]$ and Its Second Derivative $w^{\prime \prime }\left(x\right),\;x\in \left[0.5;1\right]$
Metal (*Al*)	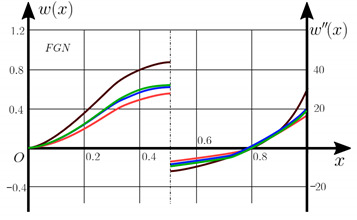
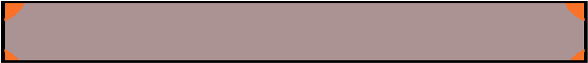	
U-PFGM	
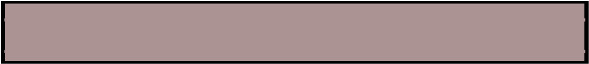	
X-PFGM	
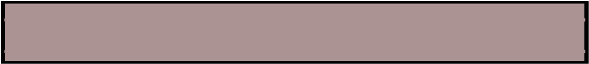	
O-PFGM	
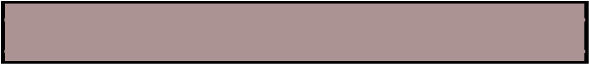	

**Table 11:** The value of the beam deflection $w\left(0.5\right)$ in the center, taking into account FGN from metal and porous structures with porosity distributions (U-PFGM, X-PFGM, O-PFGM), *λ*_1_ = 30; 50; 100, γ=0;0.5 at load *q*_0_ = 8.

*γ*	*λ*_1_ = *a/h*	Metal (*Al*)	U-PFGM	X-PFGM	O-PFGM
0	30	5.63 × 10^−1^	1.88 × 10^−1^	1.87 × 10^−1^	1.86 × 10^−1^
	50	3.33 × 10^−1^	1.76 × 10^−1^	1.76 × 10^−1^	1.75 × 10^−1^
	100	2.68 × 10^−1^	1.59 × 10^−1^	1.59 × 10^−1^	1.58 × 10^−1^
0.5	30	1.25 × 10^−1^	6.3 × 10^−2^	6.3 × 10^−2^	6.2 × 10^−2^
	50	0.5 × 10^−1^	9.4 × 10^−2^	9.4 × 10^−2^	9.3 × 10^−2^
	100	9.07 × 10^−2^	7.7 × 10^−2^	7.7 × 10^−2^	7.6 × 10^−2^

**Table 12:** The value of the beam deflection $w\left(0.5\right)$ in the center, taking into account GN from metal and porous structures with porosity distributions (U-PFGM, X-PFGM, O-PFGM), *λ*_1_ = 30; 50; 100, γ=0;0.5 at load *q*_0_ = 8.

*γ*	*λ*_1_ = *a/h*	Metal (*Al*)	U-PFGM	X-PFGM	O-PFGM
0	30	2.472 × 10^−1^	9.4 × 10^−2^	8.9 × 10^−2^	7.3 × 10^−2^
	50	3.93 × 10^−1^	3.9 × 10^−2^	3.92 × 10^−2^	3.01 × 10^−2^
	100	3.13 × 10^−1^	9.8199 × 10^−3^	9.8 × 10^−3^	1.84 × 10^−3^
0.5	30	9.4 × 10^−2^	4.64 × 10^−2^	4.5 × 10^−2^	3.509 × 10^−2^
	50	2.01 × 10^−2^	1.673 × 10^−2^	1.673 × 10^−2^	1.263 × 10^−2^
	100	1.752 × 10^−2^	4.18 × 10^−3^	4.2 × 10^−3^	4.0 × 10^−3^

**Table 13:** Influence of power coefficient k on of statics of FGNPFG beam (*λ*_1_ = 30).

k = 0.2, Γ = 0.4	k = 0.5, Γ = 0.4	k = 1, Γ = 0.4
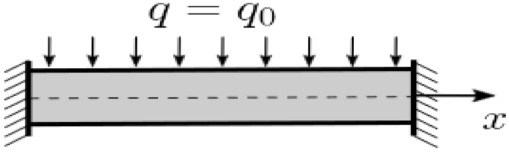 (Appendix A, (A2))		
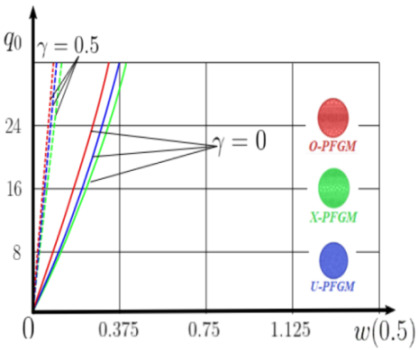	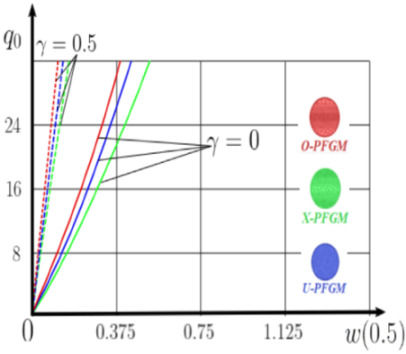	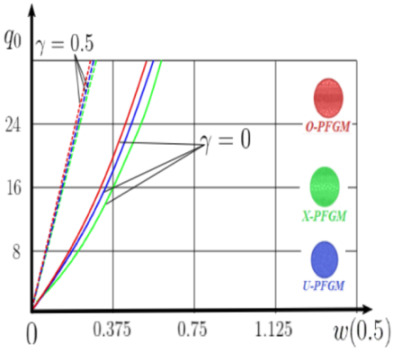
k = 0.2, Γ = 0.4	k = 0.5, Γ = 0.4	k = 1, Γ = 0.4
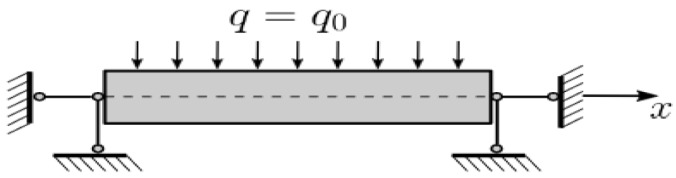 (Appendix A, (A3))		
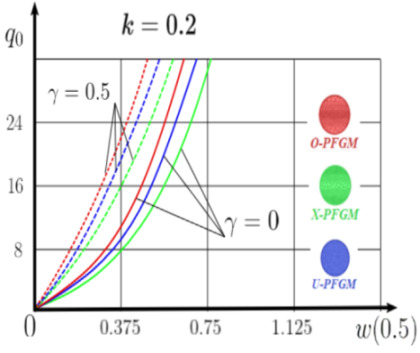	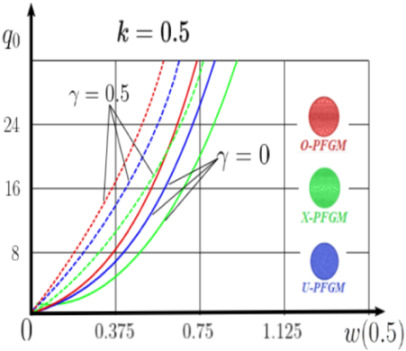	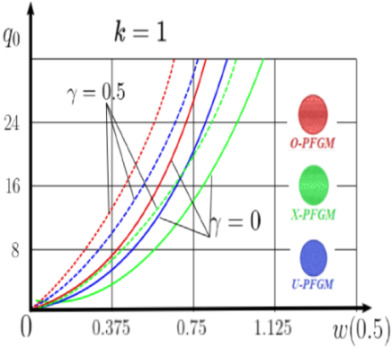

**Table 14:** The value of the load—deflection $q_{0}\left(w\left(0.5\right)\right)$ FGNPFG beam for boundary conditions (Appendix A, (A3)) depending versus the porosity index (Γ).

U-PFGM	X-PFGM	O-PFGM
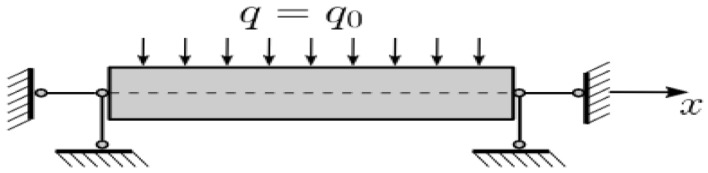 (Appendix A, (A3))		
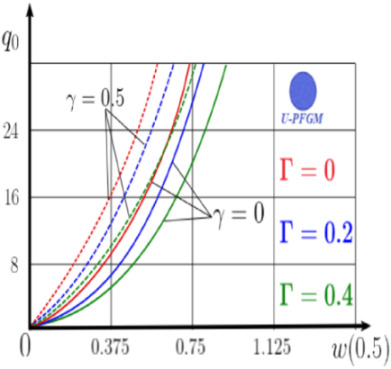	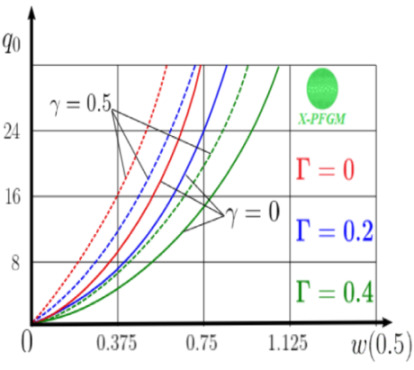	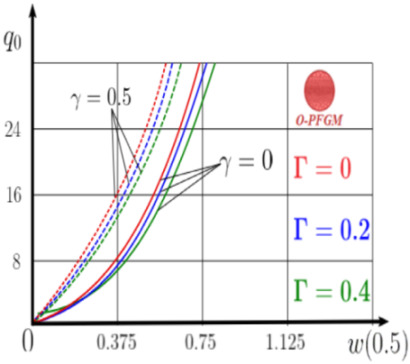

## Appendix A

The following non-dimensional PDEs with boundary and initial conditions govern of PFG nanoscaled Euler–Bernoulli beams

(A1) $$\frac{\partial }{\partial x}\left(C_{0}(x,t)\left(\frac{\partial u}{\partial x}+\frac{1}{2}{\left(\frac{\partial w}{\partial x}\right)}^{2}\right)-C_{1}(x,t)\frac{\partial^{2}w}{\partial x^{2}}\right)=\int_{-\frac{1}{2}}^{\frac{1}{2}}\rho (x,t)dz\frac{\partial^{2}u}{\partial t^{2}},\;\begin{array}{l} \frac{1}{\lambda_{1}^{2}}\frac{\partial^{2}}{\partial x^{2}}\left(C_{1}(x,t)\left(\frac{\partial u}{\partial x}+\frac{1}{2}{\left(\frac{\partial w}{\partial x}\right)}^{2}\right)-C_{2}(x,t)\frac{\partial^{2}w}{\partial x^{2}}\right)+\\ +\frac{1}{\lambda_{1}^{2}}+\frac{\partial }{\partial x}\left(C_{0}(x,t)\left(\frac{\partial u}{\partial x}+\frac{1}{2}{\left(\frac{\partial w}{\partial x}\right)}^{2}\right)-C_{1}(x,t)\frac{\partial^{2}w}{\partial x^{2}}\right)-\frac{\gamma_{}^{2}}{2\lambda_{1}^{2}}\frac{\partial^{2}}{\partial x^{2}}\left(D_{0}(x,t)\frac{\partial^{2}w}{\partial x^{2}}\right)=\\ =D_{1}(x,t)\left(\frac{\partial^{2}w}{\partial t^{2}}+\varepsilon \frac{\partial w}{\partial t}\right), \end{array}$$

1. Fixed support (both ends x=0, x=1 are fixed):

(A2)
$$w(0,t)=w(1,t)=\frac{\partial w(0,t)}{\partial x}=\frac{\partial w(1,t)}{\partial x}=u(0,t)=u(1,t)=0,$$

2. pinned support (both ends x=0,   x=1 are pinned):

(A3)
$$w(0,t)=w(1,t)=\frac{\partial^{2}w(0,t)}{\partial x^{2}}=\frac{\partial^{2}w(1,t)}{\partial x^{2}}=u(0,t)=u(1,t)=0,$$

3. one beam end is fixed, x=0 whereas the second one is pinned x=1:

(A4)
$$w(0,t)=w(1,t)=\frac{\partial w(0,t)}{\partial x}=\frac{\partial^{2}w(1,t)}{\partial x^{2}}=u(0,t)=u(1,t)=0.$$

The following initial conditions are applied:

(A5) $$w(x,0)=0,\;u(x,0)=0,\;\frac{\partial w(x,0)}{\partial t}=0,\;\frac{\partial u(x,0)}{\partial t}=0.$$

From Equations (A1)–(A5), as a special case, follow:

- Physically nonlinear PDE of PFG nanoscaled Euler–Bernoulli beam:
  (A6) $$\frac{1}{\lambda_{1}^{2}}\frac{\partial^{2}}{\partial x^{2}}\left(\left(-C_{2}(x,t)-\frac{\gamma_{}^{2}}{2}D_{0}(x,t)\right)\frac{\partial^{2}w}{\partial x^{2}}\right)+\frac{1}{\lambda_{1}^{2}}q=D_{1}(x,t)\left(\frac{\partial^{2}w}{\partial t^{2}}+\varepsilon \frac{\partial w}{\partial t}\right)$$
  1. Fixed support (both ends x=0, x=1 are fixed):
    (A7) $$w(0,t)=w(1,t)=\frac{\partial w(0,t)}{\partial x}=\frac{\partial w(1,t)}{\partial x}=0,$$
  2. pinned support (both ends x=0,   x=1 are pinned):
    (A8) $$w(0,t)=w(1,t)=\frac{\partial^{2}w(0,t)}{\partial x^{2}}=\frac{\partial^{2}w(1,t)}{\partial x^{2}}=0,$$
  3. one beam end is fixed, x=0 whereas the second one is pinned x=1:
    (A9) $$w(0,t)=w(1,t)=\frac{\partial w(0,t)}{\partial x}=\frac{\partial^{2}w(1,t)}{\partial x^{2}}=0.$$

The following initial conditions are applied:

(A10) $$w(x,0)=0,\frac{\partial w(x,0)}{\partial t}=0.$$

II. Geometrically nonlinear PDEs of PFG nanoscaled Euler–Bernoulli beam:
  (A11) $$\frac{\partial }{\partial x}\left(C_{0}(x,t)\left(\frac{\partial u}{\partial x}+\frac{1}{2}{\left(\frac{\partial w}{\partial x}\right)}^{2}\right)\right)=\int_{-\frac{1}{2}}^{\frac{1}{2}}\rho (x,t)dz\frac{\partial^{2}u}{\partial t^{2}},\;\begin{array}{l} -\frac{1}{\lambda_{1}^{2}}\frac{\partial^{2}}{\partial x^{2}}\left(C_{2}(x,t)\frac{\partial^{2}w}{\partial x^{2}}\right)+\frac{1}{\lambda_{1}^{2}}\frac{\partial }{\partial x}\left(C_{0}(x,t)\left(\frac{\partial u}{\partial x}+\frac{1}{2}{\left(\frac{\partial w}{\partial x}\right)}^{2}\right)\right)-\frac{\gamma_{}^{2}}{2\lambda_{1}^{2}}\frac{\partial^{2}}{\partial x^{2}}\left(D_{0}(x,t)\frac{\partial^{2}w}{\partial x^{2}}\right)=\\ =D_{1}(x,t)\left(\frac{\partial^{2}w}{\partial t^{2}}+\varepsilon \frac{\partial w}{\partial t}\right), \end{array}$$
  1. Fixed support (both ends x=0, x=1 are fixed):
    (A12) $$w(0,t)=w(1,t)=\frac{\partial w(0,t)}{\partial x}=\frac{\partial w(1,t)}{\partial x}=u(0,t)=u(1,t)=0,$$
  2. pinned support (both ends x=0,   x=1 are pinned):
    (A13) $$w(0,t)=w(1,t)=\frac{\partial^{2}w(0,t)}{\partial x^{2}}=\frac{\partial^{2}w(1,t)}{\partial x^{2}}=u(0,t)=u(1,t)=0,$$
  3. one beam end is fixed, x=0 whereas the second one is pinned x=1:
    (A14) $$w(0,t)=w(1,t)=\frac{\partial w(0,t)}{\partial x}=\frac{\partial^{2}w(1,t)}{\partial x^{2}}=u(0,t)=u(1,t)=0.$$

The following initial conditions are applied:

(A15) $$w(x,0)=0,u(x,0)=0,\frac{\partial w(x,0)}{\partial t}=0,\frac{\partial u(x,0)}{\partial t}=0.$$
